# Respiratory Depression Following Concomitant Infusion of Remimazolam and Remifentanil Using Targeted Effect-Site Concentrations: A Randomized Controlled Trial

**DOI:** 10.3390/medicina62050940

**Published:** 2026-05-12

**Authors:** Ha Yeon Kim, Sang Kee Min, Jee Hwan Moon, Hyeongjin Kwak, Soo Jung Park

**Affiliations:** 1 Department of Anesthesiology and Pain Medicine, Ajou University School of Medicine, 164 Worldcup-ro, Youngtong-gu, Suwon-si 16499, Gyeonggi-do, Republic of Korea; hayeon@aumc.ac.kr (H.Y.K.); anesmin@aumc.ac.kr (S.K.M.); abrasax90@naver.com (J.H.M.); bluejin327@gmail.com (H.K.); 2 Department of Medicine, Ajou University School of Medicine, 164 Worldcup-ro, Youngtong-gu, Suwon-si 16499, Gyeonggi-do, Republic of Korea; 3 Department of Anesthesiology and Pain Medicine, Anesthesia and Pain Research Institute, Yonsei University College of Medicine, 50-1 Yonsei-ro, Seodaemun-gu, Seoul 03722, Republic of Korea

**Keywords:** remimazolam, remifentanil, respiratory depression, target-controlled infusion, sedation

## Abstract

*Background and Objectives:* Remimazolam and remifentanil are ultra-short-acting agents that are used for sedation and analgesia, respectively. Their combined effect on respiratory function is unclear. We evaluated whether co-administration produced dose-dependent respiratory depression and loss of consciousness (LOC) preceded oxygen desaturation. *Materials and Methods:* A randomized, double-blind trial was conducted from May to July 2024. Female patients (20–65 years; *n* = 108; American Society of Anesthesiologists physical status I–II) undergoing elective gynecological surgery were selected. Patients received remifentanil via target-controlled infusion (TCI) at effect-site concentrations (C_e_) of 1.0, 1.5, or 2.0 ng/mL (Groups 1.0, 1.5, and 2.0) combined with a fixed C_e_ of 500 ng/mL remimazolam. Respiratory variables, timing of LOC, bispectral index, and adverse events were recorded. *Results:* Respiratory depression increased in a dose-dependent manner. Jaw thrust was required in 52.8% of Group 1.0 and 91.7% of Group 2.0 (*p* < 0.001). The need for 100% oxygen increased from 30.6% to 69.4% (*p* = 0.001). Minute ventilation decreased only in Group 2.0 (*p* = 0.008). Involuntary movements were frequent in Group 1.0 (*p* = 0.005). *Conclusions:* Remimazolam–remifentanil co-administration via TCI induced dose-dependent respiratory depression and pre-LOC desaturation. Therefore, continuous monitoring and careful titration are essential.

## 1. Introduction

Remifentanil and remimazolam are ultra-short-acting agents that are commonly used for anesthesia. Remifentanil, a μ-opioid receptor agonist, provides potent analgesia during surgery or painful procedures. Unlike other opioids, it exhibits the unique feature of rapid offset owing to its rapid degradation by abundant, non-specific esterases in the plasma [1,2]. Remimazolam, a novel gamma-aminobutyric acid (GABA) receptor agonist, is used for anesthesia induction and hypnosis. It also undergoes rapid metabolism by nonspecific esterases, resulting in both rapid onset and offset of action [3,4]. Given these pharmacokinetic properties, the concomitant use of remimazolam and remifentanil is considered an optimal anesthetic strategy, enabling precise titration of both hypnotic and analgesic effects, while ensuring rapid postoperative recovery.

Target-controlled infusion (TCI) offers the advantage of achieving a stable effect-site concentration without the risk of overshoot or overdose [5]. Several pharmacokinetic/pharmacodynamic (PK/PD) models have been established for remifentanil, and TCI-based continuous infusion is widely practiced in clinical anesthesia [6,7,8,9]. The recommendation of the current manufacturer for remimazolam involves an initial administration at 6.0–12.0 mg/kg/h until loss of consciousness (LOC), followed by a maintenance infusion of 1.0–2.0 mg/kg/h. However, recent research suggests that this continuous infusion strategy may lead to high peak plasma concentrations and requires nearly 1 h to achieve steady-state levels. Although several TCI models have already been developed [10,11,12,13], these findings still underscore the need to explore the applicability of TCI in remimazolam administration [14,15].

Although multiple studies have indicated that higher benzodiazepine concentrations are associated with an increased risk of respiratory depression [16,17,18], data on remimazolam-induced respiratory depression are scarce. Similarly, remifentanil, like other opioids, is known to produce dose-dependent respiratory depression [19,20]. A previous study conducted at our center demonstrated that remimazolam administered via TCI resulted in dose-dependent respiratory depression, similar to other benzodiazepines, with higher effect-site concentrations requiring more frequent respiratory support [21]. Hence, in this study, we aimed to investigate the extent of respiratory depression when remimazolam and remifentanil were co-administered using TCI.

## 2. Materials and Methods

### 2.1. Ethics

This was a single-center, prospective, double-blind, randomized trial. Ethical approval for this study (AJOUIRB-IV-2023-249) was granted by the Institutional Review Board of Ajou University, School of Medicine, Suwon, Republic of Korea (Chairperson Prof. Jai Sung Noh) on 26 May 2023. The study was also registered with the Clinical Research Information Service (CRIS; KCT0009178), a primary registry in the WHO International Clinical Trials Registry Platform, on 16 February 2024. All procedures were conducted in accordance with the ethical principles of the Declaration of Helsinki (2013 revision) [22]. Written informed consent was obtained from all participants after they were given adequate time for consideration.

### 2.2. Study Population and Allocation

A total of 108 patients aged 20–65 years, classified as American Society of Anesthesiologists (ASA) physical status I or II and scheduled for gynecologic surgery, were enrolled in the study. The exclusion criteria were a body mass index ≥ 30 kg/m^2^, Mallampati class III or IV airway, obstructive sleep apnea, diagnosed pulmonary disease, upper airway infection within the past 3 weeks, liver disease (as defined by aspartate aminotransferase or alanine aminotransferase > 100 U/L), or renal impairment (serum creatinine ≥ 177 µmol/L).

Participants were randomized into three groups (1:1:1 ratio) based on the target effect-site concentration (C_e_) of remifentanil, using TCI at 1.0, 1.5, and 2.0 ng/mL. Randomization was conducted using a computer-generated sequence in Microsoft Excel 2007 (Microsoft, Redmond, WA, USA). To maintain blinding, group allocation was concealed and revealed only to an anesthesiologist responsible for operating the syringe pump immediately before surgery. All outcome assessors remained unaware of patients’ group assignments throughout the study.

No premedication was administered prior to the surgery. Upon arrival at the operating room, a 20-G intravenous cannula was inserted into the forearm or dorsum of the hand and connected to a three-way stopcock. Standard monitoring was applied, including electrocardiography (ECG), pulse oximetry, bispectral index (BIS), and non-invasive blood pressure (NIBP) measurement. Before anesthesia induction, baseline respiratory mechanics, including tidal volume (TV), end-tidal carbon dioxide (EtCO_2_), respiratory rate (RR), and minute ventilation (MV), were measured using a tightly fitted facial mask with 5 L/min of fresh gas flow at a fraction of inspired oxygen (FiO_2_) of 21%. Baseline hemodynamic variables including heart rate (HR), mean arterial pressure (MAP), and BIS were recorded. Remimazolam and remifentanil infusions were initiated after the baseline measurements were completed.

Study drugs were prepared by an independent nurse not involved in data collection. Remimazolam (Byfavo Inj., Hana Pharma Co. Ltd., Seoul, Republic of Korea) was prepared by diluting 50 mg of the drug with 50 mL of 0.9% saline, resulting in a final concentration of 1 mg/mL. It was administered via TCI to achieve a target C_e_ of 500 ng/mL in all patients based on previous findings indicating minimal respiratory support requirements at this concentration [21]. TCI was conducted using remimazolam PK/PD software (Asan Pump, ver 2.1.3, Bionet Co., Ltd., Seoul, Republic of Korea), using the model of Schuttler et al. [12] and a compatible syringe pump (Pilote Anesthesie 2 IS, Fresenius Vial, Le Grand Chemin, Brezins, France), which was connected to a laptop computer via a USB-serial-port connection. The maximum infusion rate for remimazolam was set at 1200 mL/h.

Remifentanil (Ultiva; GlaxoSmithKline, Parma, Italy) was prepared by diluting 2.0 mg of the drug in 50 mL of 0.9% saline to achieve a final concentration of 40 µg/mL. The solution was delivered using a TCI pump (Orchestra; Fresenius Vial) using the pharmacokinetic model of Minto et al. [6], with the maximum infusion rate set at 200 mL/h in all groups. Patients were randomized into three groups based on the target C_e_ of remifentanil. To conceal the infusion rate of drugs, infusion syringes and pumps were covered with opaque material.

Respiratory depression was defined as decreased oxygen saturation (SpO_2_). Interventions were applied in a stepwise manner based on SpO_2_ thresholds: a jaw-thrust maneuver (chin lift/neck extension) was used when SpO_2_ decreased to below 97%, FiO_2_ was increased to 100% when SpO_2_ decreased to below 93%, and assisted ventilation was initiated when SpO_2_ decreased to below 90%. All monitoring and interventions were performed by an attending anesthesiologist at the bedside throughout the 10 min observation. The duration of the observation period was determined based on pharmacokinetic simulations, indicating that remimazolam and remifentanil reached their target C_e_ within approximately 3 min. Therefore, a 10 min monitoring period was selected to allow sufficient time to capture the pharmacodynamic effects and potential respiratory events. No patient was intentionally maintained in a hypoxemic state; each intervention was applied without delay, and all desaturation episodes were promptly reversed.

During remimazolam and remifentanil administration, the level of consciousness was assessed every 10 s using the Modified Observer’s Assessment of Alertness/Sedation (MOAA/S) scale, concurrently with BIS monitoring. LOC was defined as a MOAA/S score ≤ 2. After LOC was confirmed, re-awakening, defined as an MOAA/S score ≥ 3 maintained for at least 10 s, was assessed at 1 min intervals. BIS monitoring was performed to obtain additional objective data on sedation depth. At the end of the 10 min study period, 100% oxygen was administered and neuromuscular blockade was induced to facilitate endotracheal intubation according to standard protocols.

### 2.3. Definitions and Measurements

Demographic data including sex, age, height, and weight were collected. The primary endpoints were the frequency and timing of three specific respiratory support interventions: jaw-thrust, administration of 100% FiO_2_, and assisted ventilation. Secondary endpoints included respiratory variables (TV, RR, MV, and EtCO_2_), hemodynamic parameters (MAP and HR), and depth of hypnosis assessed using the BIS. These variables were measured at baseline (0 min), 5 min, and 10 min, whereas the BIS was additionally assessed at the time of LOC. Intergroup and intragroup comparisons were conducted for these repeated-measure variables. Adverse events, such as re-awakening, vasopressor use (MAP < 65 mmHg), hiccups, and involuntary movements, were also compared between the groups.

### 2.4. Statistical Analysis

The sample size was determined based on preliminary data from a pilot study that assessed the incidence of respiratory depression at a target remimazolam C_e_ of 500 ng/mL combined with varying remifentanil C_e_ levels. The primary outcome was the incidence of SpO_2_ desaturation below 93%, which occurred in 15% of the patients who received remimazolam at 500 ng/mL and remifentanil at 1.0 ng/mL. For comparisons across the three remifentanil C_e_ targets (1.0, 1.5, and 2.0 ng/mL), the calculated effect size (Cohen’s *v*) was 0.31. To detect significant differences in SpO_2_ desaturation across the groups, a minimum of 33 patients per group was required (α = 0.05, power = 0.8). Allowing for a 10% dropout rate, the final sample size was set at 36 patients per group, for a total of 108 patients.

All continuous variables are expressed as mean ± SD or median with interquartile range (IQR), as appropriate. Categorical variables were compared using the chi-squared or Fisher’s exact tests. Continuous variables were analyzed using one-way analysis of variance or the Kruskal–Wallis test, depending on the distribution determined by the normality test. When significant differences were found, the Bonferroni correction was applied for post hoc multiple comparisons. Absolute risk differences and relative risks with corresponding 95% confidence intervals were calculated to quantify effect sizes for the primary outcomes. Repeated-measures continuous variables were analyzed using a linear mixed-effects model. Timepoint and group interactions were determined using post hoc tests. Statistical significance was set at *p* < 0.05.

## 3. Results

**Figure 1:**
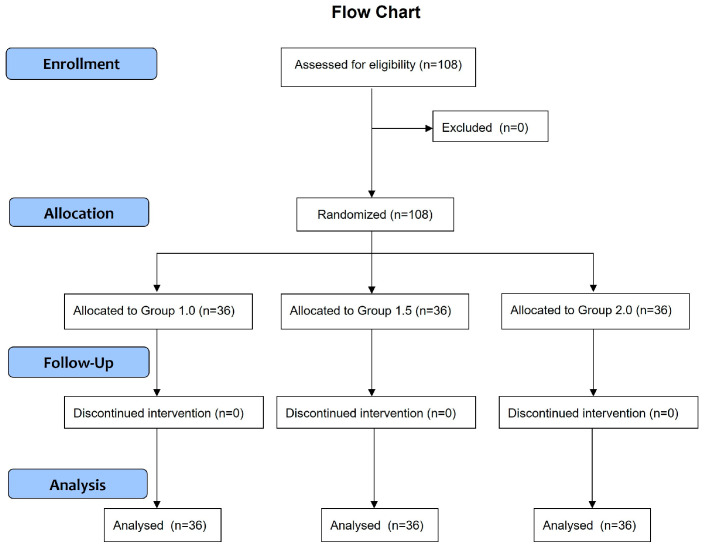
Flow chart. CONSORT diagram showing patient enrollment, randomization, allocation, and analysis.

A total of 108 patients were enrolled between 30 May and 17 July 2024, and none were lost to follow-up. The CONSORT flowchart is shown in Figure 1. The demographic data are presented in Table 1. Baseline demographic characteristics were similar among the three groups. Patients who developed oxygen desaturation (SpO_2_ < 90%) during the 10 min observation period received 100% FiO_2_ and manual ventilation, which effectively restored SpO_2_ to >98%.

**Figure 2:**
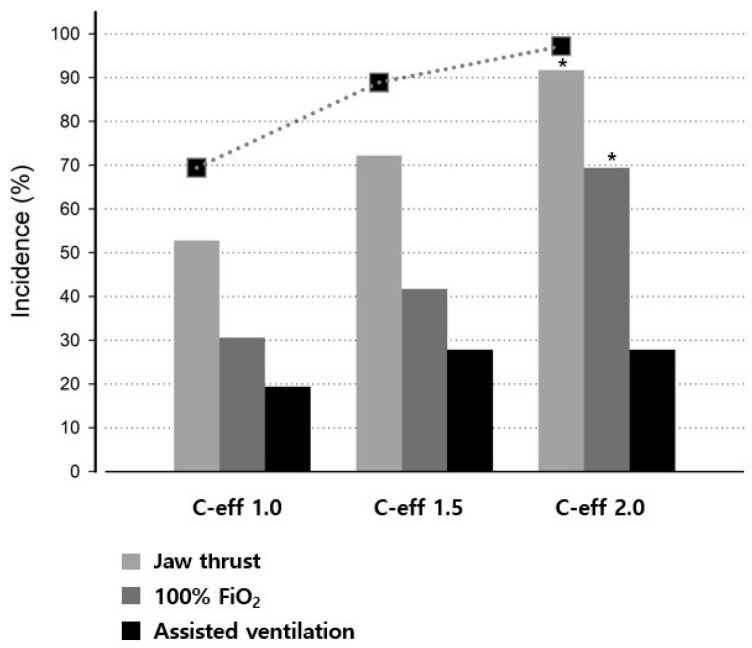
Incidence of respiratory interventions across remifentanil concentrations. Incidence (%) of respiratory interventions during co-infusion of remimazolam (C_e_ 500 ng/mL) with remifentanil at effect-site concentrations of 1.0, 1.5, and 2.0 ng/mL (Groups 1.0, 1.5, and 2.0). Bars indicate jaw thrust (light gray), 100% FiO_2_ administration (dark gray), and assisted ventilation (black), which were applied at predefined thresholds (SpO_2_ < 97%, <93%, and <90%, respectively). The dotted line with squares indicates the cumulative incidence of any intervention per group. Data are presented as n (%). * *p* < 0.05 vs. Groups 1.0 and 1.5.

Of the patients that experienced desaturation, the time to jaw-thrust (SpO_2_ < 97%) was 68–302 s following the initiation of remimazolam and remifentanil TCI, with a mean onset time of 133 ± 44.5 s. A higher target C_e_ for remifentanil was associated with more frequent respiratory interventions (Table 2). The incidence of jaw thrust was higher in Group 2.0 than in Group 1.0 (*p* < 0.001) and Group 1.5 (*p* = 0.032). Similarly, the incidence of 100% FiO_2_ administration was higher in Group 2.0 than in Groups 1.0 (*p* = 0.001) and 1.5 (*p* = 0.016). However, the incidence of assisted ventilation did not differ substantially among the three groups (Figure 2). These episodes of respiratory depression occurred after LOC in all cases, except for one patient in Group 1.0. Among the 16 patients who did not achieve LOC, three developed respiratory depression requiring jaw-thrust.

**Figure 3:**
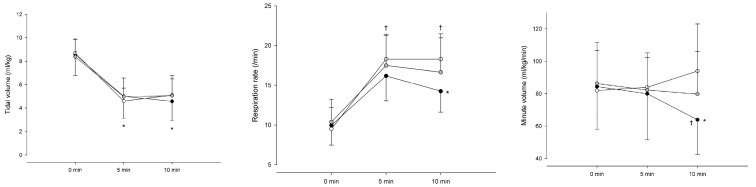
Respiratory variables over time. (**a**) Tidal volume, (**b**) respiratory rate, and (**c**) minute ventilation at 0, 5, and 10 min during co-infusion of remimazolam (C_e_ 500 ng/mL) with remifentanil at effect-site concentrations of 1.0, 1.5, and 2.0 ng/mL. Symbols represent Group 1.0 (black circles), Group 1.5 (gray circles), and Group 2.0 (white circles). Values are mean ± SD. * *p* < 0.05 vs. baseline; † *p* < 0.05 between groups.

Regarding changes in respiration patterns, intergroup comparisons of TV differences among the three groups showed no significant differences at any time point. However, intragroup comparisons indicated that TV decreased at 5 and 10 min compared to that at baseline (0 min) in all groups (*p* < 0.001) (Figure 3a). For RR, no intergroup differences were observed at 0 and 5 min; however, at 10 min, Group 2.0 had a lower RR than Group 1.0 (*p* < 0.001). Intragroup comparisons indicated that RR increased at both 5 and 10 min relative to that at baseline in all groups (*p* < 0.001) (Figure 3b). The intergroup comparison indicated that MV at 10 min was lower in Group 2.0 than in Groups 1.0 (*p* < 0.001) and 1.5 (*p* = 0.03). In the intragroup analysis, only Group 2.0 demonstrated a decrease in MV at 10 min compared to that at baseline (*p* = 0.008) (Figure 3c). For EtCO_2_, intragroup analysis indicated a decrease in Group 1.5 at 5 min (*p* = 0.05) and 10 min (*p* = 0.011) compared to that at baseline.

**Figure 4:**
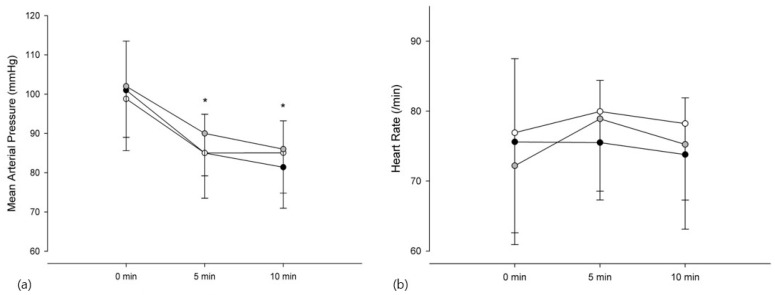
Hemodynamic variables over time. (**a**) Mean arterial pressure and (**b**) heart rate at 0, 5, and 10 min during co-infusion of remimazolam (C_e_ 500 ng/mL) with remifentanil at effect-site concentrations of 1.0, 1.5, and 2.0 ng/mL. Symbols represent Group 1.0 (black circles), Group 1.5 (gray circles), and Group 2.0 (white circles). Values are mean ± SD. * *p* < 0.05 vs. baseline.

**Figure 5:**
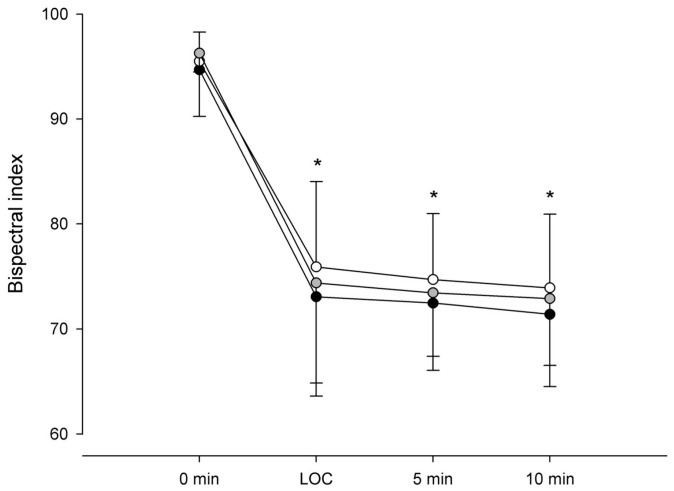
Bispectral index at key timepoints. Bispectral index at baseline, loss of consciousness, 5 min, and 10 min during co-infusion of remimazolam (C_e_ 500 ng/mL) with remifentanil at effect-site concentrations of 1.0, 1.5, and 2.0 ng/mL. Symbols represent Group 1.0 (black circles), Group 1.5 (gray circles), and Group 2.0 (white circles). Values are mean ± SD. * *p* < 0.05 vs. baseline.

For hemodynamic variables, inter-group comparisons of MAP differences among the three groups did not differ at any time point. By contrast, intragroup comparisons indicated that MAP decreased at 5 and 10 min compared to that at baseline in all groups (*p* < 0.001) (Figure 4a). HR was similar between and within all groups at any time point (Figure 4b). A higher target C_e_ concentration of remifentanil was associated with an increased incidence of LOC (Table 3). LOC occurred more frequently in Groups 1.5 (*p* = 0.04) and 2.0 (*p* = 0.001) than in Group 1.0. BIS values did not differ between the groups at baseline, LOC, 5 min, and 10 min; however, intragroup comparisons indicated that BIS at LOC, 5 min, and 10 min was lower than that at baseline in all groups (*p* < 0.001) (Figure 5). The incidence of re-awakening, vasopressor use for hypotension, and hiccups was similar among the groups, but involuntary movement occurred more often in Group 2.0 than in Group 1.0 (*p* = 0.005) (Table 4).

## 4. Discussion

In this study, the effects of remifentanil on respiratory depression when administered in combination with remimazolam at a fixed concentration of 500 ng/mL were evaluated. As the remifentanil C_e_ increased from 1.0 to 2.0 ng/mL, the incidence of respiratory depression rose substantially. In most cases, LOC preceded respiratory depression; however, in four patients, respiratory depression occurred either prior to or in the absence of LOC.

At the highest remifentanil concentration (Group 2.0), the incidence of jaw-thrust, administration of 100% oxygen, and assisted ventilation were 91.7%, 69.4%, and 27.8%, respectively. In a previous study, remimazolam was administered alone at the same target C_e_ of 500 ng/mL; the corresponding incidence rates were 61.1%, 22.2%, and 2.8%, respectively [21]. Although direct comparisons between studies have inherent limitations, the substantial increase observed in the current study suggests a possible additive respiratory effect of remifentanil and remimazolam to promote respiratory depression. This finding is consistent with previous evidence that opioids and benzodiazepines exert additive or synergistic respiratory depressive effects via distinct but convergent pathways within brainstem respiratory centers [23,24,25,26].

In previous studies involving sedative agents alone, LOC consistently preceded respiratory depression [21,27,28]. However, in the current study, which combined remimazolam with remifentanil, four patients exhibited respiratory depression before or in the absence of LOC. This discrepancy may suggest PD dissociation between the respiratory effects of sedatives and opioids. Sedatives suppress cortical activity through GABA*_A_* receptor modulation, leading to sedation and LOC. In clinical studies, respiratory depression has typically been observed only at high doses or deep levels of sedation [4,27]. By contrast, opioids act more directly on brainstem respiratory centers as μ-opioid receptor agonists, potentially inducing respiratory depression independent of cortical suppression [29]. In addition, the faster onset of remifentanil than remimazolam may result in respiratory depression preceding the hypnotic effect of remimazolam. Therefore, both PK and PD factors of remimazolam and remifentanil caused respiratory depression even in the absence of LOC in some patients. Further investigations are required to clarify this dissociation.

Benzodiazepine sedatives generally reduce TV while increasing RR [21,30,31], whereas opioids decrease RR with relative preservation of TV [29,32,33]. In the current study, co-administration of remimazolam and remifentanil resulted in a pattern consistent with compensatory shallow breathing, characterized by a decrease in TV accompanied by an increase in RR. This pattern likely reflects a compensatory mechanism to maintain MV despite reduced TV. However, as the C_e_ of remifentanil increased, this compensatory RR response was attenuated, leading to a reduction in MV at the highest concentration. These findings suggest that while remimazolam may predominantly induce shallow breathing, increasing remifentanil concentrations may blunt the compensatory respiratory response through its effects on brainstem respiratory control, resulting in dose-dependent ventilatory impairment.

A previous study [21] reported a jaw-thrust incidence of 61.1% at a target C_e_ of 500 ng/mL of remimazolam alone; the current study observed a slightly lower incidence of 52.8% in Group 1.0, despite the co-administration of remifentanil. Although the combination with remifentanil is typically expected to increase the risk of respiratory depression, this unexpected reduction may be attributable to differences in patient positioning during sedation. In a previous study [21], a jelly doughnut-shaped gel pillow was used, whereas in the current study, this was replaced with a disposable pillow of a slightly greater height. This difference in head and neck support may have influenced the upper airway patency, contributing to the lower incidence of jaw thrust observed. However, direct comparison between these two studies is limited, as they were conducted at different times and in different patient cohorts, and random sampling variability and other unmeasured between-study factors cannot be excluded. Therefore, we do not draw a firm mechanistic conclusion from this cross-study observation, but rather highlight the need for standardized positioning in future studies of sedation-associated respiratory depression.

This study had some limitations. First, all participants were female, which limits the generalizability of the findings to the male population. Although differences in lean body mass between the sexes are generally modest, recent studies have suggested that the effective dose of remimazolam may differ between men and women based on PK and PD modeling [10,12,34]. These findings emphasize the need for further investigation in male populations to assess the potential sex-based variability in drug responses. Second, although baseline characteristics were well balanced across groups in this randomized controlled trial, the range of collected variables was limited and multivariable adjustment was not performed; therefore, residual confounding by unmeasured factors cannot be completely excluded. Third, this study did not include a wide range of remifentanil and remimazolam concentrations, thereby restricting the analysis to a narrow C_e_ range. Broader concentration ranges should be evaluated in future studies to elucidate respiratory dynamics. Fourth, we cannot completely exclude the possibility of air leakage associated with the use of a facial mask for TV measurements. Fifth, arterial CO_2_ measurements were not performed because placement of an arterial line solely for research purposes was not justified in this patient population without a clinical indication. Sixth, because this study was conducted in a controlled setting without procedural or surgical stimuli, further research is needed to assess the respiratory dynamics under various clinical conditions. Finally, because the MOAA/S scale was assessed every 10 s, repeated verbal or tactile stimulation may have introduced minimal interference with sedation depth, although each assessment lasted only a few seconds and was unlikely to have altered the outcomes substantially.

## 5. Conclusions

In conclusion, we demonstrated that the co-administration of remimazolam and remifentanil using TCI resulted in dose-dependent respiratory depression, with higher C_e_ associated with an increased frequency of respiratory interventions. Although respiratory depression predominantly occurred after LOC, some cases of pre-LOC desaturation were observed, highlighting the importance of vigilant respiratory monitoring. Careful dose titrations of both agents and proactive airway management strategies are essential. These findings provide clinically relevant evidence to guide the safe co-administration of remimazolam and remifentanil and establish foundational data for future development of remimazolam TCI protocols.

## Figures and Tables

**Table 1:** Demographic data.

Variable	Group 1.0 (*n* = 36)	Group 1.5 (*n* = 36)	Group 2.0 (*n* = 36)
Age, year	44 ± 14.4	44 ± 14.4	46 ± 12.8
Body weight, kg	61 ± 9.8	62 ± 10.4	58 ± 10.4
Height, cm	159 ± 6.4	159 ± 5.5	158 ± 6.7
Body mass index, kg/m^2^	24 ± 3.7	24 ± 4.2	23 ± 4.1

Data were presented as mean ± SD. The target effect-site concentrations of remifentanil were 1.0, 1.5 and 2.0 ng/mL for Groups 1.0, 1.5, and 2.0, respectively, with concomitant remimazolam TCI at a fixed target of 500 ng/mL in all groups.

**Table 2:** Incidence of respiratory interventions according to remifentanil effect-site concentration during co-administration with remimazolam at 500 ng/mL.

**(A) Incidence of Respiratory Interventions**				
**Intervention**	**Group 1.0** **(** ***n*** ** = 36)**	**Group 1.5** **(** ***n*** ** = 36)**	**Group 2.0** **(** ***n*** ** = 36)**	***p*** ** -Value**
Jaw thrust, n (%) Onset, s	19 (52.8) 134 ± 44.1	26 (72.2) 142 ± 51.9	33 (91.7) * 127 ± 38.4	<0.001 0.411
100% FiO_2_, n (%)	11 (30.6)	15 (41.7)	25 (69.4) *	0.001
Assisted ventilation, n (%)	7 (19.4)	10 (27.8)	10 (27.8)	0.416
**(B) Effect Size Estimates (Reference: Group 1.0)**				
**Outcome**	**Comparison**	**Risk Difference (95% CI)**	**Relative Risk (95% CI)**	
Jaw thrust	1.5 vs. 1.0	+19.4% (−3.0 to 41.8)	1.37 (0.95–1.98)	
	2.0 vs. 1.0	+38.9% (18.9 to 58.9)	1.74 (1.26–2.40)	
100% FiO_2_	1.5 vs. 1.0	+11.1% (−11.6 to 33.8)	1.36 (0.73–2.55)	
	2.0 vs. 1.0	+38.9% (17.6 to 60.2)	2.27 (1.33–3.89)	
Assisted ventilation	1.5 vs. 1.0	+8.3% (−11.9 to 28.5)	1.43 (0.61–3.34)	
	2.0 vs. 1.0	+8.3% (−11.9 to 28.5)	1.43 (0.61–3.34)	

CI: confidence interval. Data were presented as incidence (number of patients, %), and time data were mean ± standard deviation. The target effect-site concentrations of remifentanil were 1.0, 1.5, and 2.0 ng/mL for Groups 1.0, 1.5, and 2.0, respectively, with concomitant remimazolam TCI at a fixed target of 500 ng/mL in all groups. * *p* < 0.05 vs. Groups 1.0 and 1.5.

**Table 3:** Incidence and time to loss of consciousness according to remifentanil effect-site concentration during co-administration with remimazolam at 500 ng mL^−1^.

Loss of Consciousness	Group 1.0 (*n* = 36)	Group 1.5 (*n* = 36)	Group 2.0 (*n* = 36)	*p*-Value
Incidence, n (%)	25 (69.4)	32 (88.9) *	35 (97.2) *	0.001
Time, seconds	93 (70–100)	100 (80–110)	81 (60–90)	0.140

Data are presented as numbers (%) or medians (25th–75th IQR). The target effect-site concentrations of remifentanil were 1.0, 1.5, and 2.0 ng/mL for Groups 1.0, 1.5, and 2.0, respectively, with concomitant remimazolam TCI at a fixed target of 500 ng/mL in all groups. * *p* < 0.05 vs. Group 1.0.

**Table 4:** Incidence of other events according to remifentanil effect-site concentration during co-administration with remimazolam at 500 ng mL^−1^.

Event	Group 1.0 (*n* = 36)	Group 1.5 (*n* = 36)	Group 2.0 (*n* = 36)	*p*-Value
Re-awake; once	3 (8.6)	6 (17.1)	4 (11.4)	0.709
Re-awake; more than once	3 (8.3)	1 (2.9)	2 (5.7)	0.609
Vasopressor	1 (2.9)	1 (2.9)	0 (0.0)	0.384
Involuntary movement	14 (40.0)	9 (25.0)	4 (11.1) *	0.005
Hiccup	1 (2.9)	5 (14.5)	1 (2.9)	>0.999

Data are expressed as numbers (%). The target effect-site concentrations of remifentanil were 1.0, 1.5 and 2.0 ng mL^−1^ for Groups 1.0, 1.5, and 2.0, respectively, with concomitant remimazolam TCI at a fixed target of 500 ng/mL in all groups. * *p* < 0.05 vs. Group 1.0.

## Data Availability

The datasets generated and/or analyzed during the current study are available from the corresponding author (Soo Jung Park) upon reasonable request.

## Abbreviations

The following abbreviations are used in this manuscript:

GABA	Gamma-aminobutyric acid
LOC	Loss of consciousness
TCI	Target-controlled infusion
PK	Pharmacokinetic
PD	Pharmacodynamic
ASA	American Society of Anesthesiologists
ECG	Electrocardiography
BIS	Bispectral index
NIBP	Non-invasive blood pressure
C_e_	Effect-site concentration
TV	Tidal volume
RR	Respiratory rate
MV	Minute ventilation
MAP	Mean arterial pressure
EtCO_2_	End-tidal carbon dioxide
HR	Heart rate
SpO2	Oxygen saturation
MOAA/S	Modified Observer’s Assessment of Alertness/Sedation
IQR	Interquartile range
FiO_2_	Fraction of inspired oxygen
